# Experiments on Socio-Technical Systems: The Problem of Control

**DOI:** 10.1007/s11948-015-9634-4

**Published:** 2015-02-22

**Authors:** Peter Kroes

**Affiliations:** Department of Philosophy/TPM, Delft University of Technology, Jaffalaan 5, 2628 BX Delft, The Netherlands

**Keywords:** Socio-technical systems, Experiments, Natural sciences, Social sciences, Control, Technological innovations

## Abstract

My aim is to question whether the introduction of new technologies in society may be considered to be genuine experiments. I will argue that they are not, at least not in the sense in which the notion of experiment is being used in the natural and social sciences. If the introduction of a new technology in society is interpreted as an experiment, then we are dealing with a notion of experiment that differs in an important respect from the notion of experiment as used in the natural and social sciences. This difference shows itself most prominently when the functioning of the new technological system is not only dependent on technological hardware but also on social ‘software’, that is, on social institutions such as appropriate laws, and actions of operators of the new technological system. In those cases we are not dealing with ‘simply’ the introduction of a new technology, but with the introduction of a new socio-technical system. I will argue that if the introduction of a new socio-technical system is considered to be an experiment, then the relation between the experimenter and the system on which the experiment is performed differs significantly from the relation in traditional experiments in the natural and social sciences. In the latter experiments it is assumed that the experimenter is not part of the experimental system and is able to intervene in and control the experimental system from the outside. With regard to the introduction of new socio-technical systems the idea that there is an experimenter outside the socio-technical system who intervenes in and controls that system becomes problematic. From that perspective we are dealing with a different kind of experiment.

## Introduction

Following Martin and Schinzinger (1989) the introduction of new technologies in society has often been interpreted as performing experiments on society. One of the main reasons for doing so is that the effects of new technologies on society are usually hard to predict. In the absence of reliable knowledge about these effects, an experimental approach may be appropriate for at least the following two reasons. First, experiments are performed to learn and so treating technological innovations as experiments calls for a close monitoring of the societal effects of a new technology; without a close monitoring nothing will be learned about the possible effects of new technologies. But apart from learning through close monitoring the experimental approach is of crucial importance for a second reason. Experiments are usually performed under controlled conditions such that it is possible to stop an experiment. If the monitored effects of the introduction of a new technology are, for whatever reasons (technical, social, moral, economic etcetera), not desirable, and if the introduction of the new technology is set up as a real experiment, then it should be possible in principle to stop the experiment.

If following Martin and Schinzinger the introduction of a new technology is considered to be a genuine experiment on society, then this raises interesting ethical questions. For instance, the question arises whether, by analogy to medical experiments on human beings, the notion of informed consent may be applied to these experiments on society, since human beings are involved [see (Martin and Schinzinger 1989, Ch. 3)]. It is not my intention to enter here into a discussion of these ethical issues. In my opinion, a proper discussion of these ethical issues requires first of all a closer examination of the idea that the introduction of new technologies may be looked upon as real experiments on society. That is what I intend to do in this paper.

My aim is to question whether the introduction of new technologies into society may be considered to be genuine experiments. I will argue that they are not, at least not in the sense in which the notion of experiment is being used in the natural and social sciences. If the introduction of a new technology in society is interpreted as an experiment, then we are dealing with a notion of experiment that differs in an important respect from the notion of experiment as used in the natural and social sciences. This difference shows itself most prominently when the functioning of the new technological system is not only dependent on technological hardware but also on social ‘software’, that is, on social institutions such as appropriate laws, and actions of operators of the new technological system. In those cases we are not dealing with ‘simply’ the introduction of a new technology, but with the introduction of a new socio-technical system. I will argue that if the introduction of a new socio-technical system is considered to be an experiment, then the relation between the experimenter and the system on which the experiment is performed differs significantly from the relation in traditional experiments in the natural and social sciences. In the latter experiments it is assumed that the experimenter is not part of the experimental system and is able to intervene in and control the experimental system from the outside. With regard to the introduction of new socio-technical systems the idea that there is an experimenter outside the socio-technical system who intervenes in and controls that system becomes problematic. From that perspective we are then dealing with different kinds of experiments. In my opinion, discussions about the possibility or desirability of the application of the traditional moral requirement of informed consent to technological innovations as experiments on society will have to take into account this difference in the kinds of experiments.

## Experiments and Control in the Natural Sciences

I will start with a brief look at the various kinds of experiments performed in the natural sciences. Depending on so to speak the *locus* of the experiment we may distinguish between thought experiments which take place in the mind, computational/simulation experiments in computers and experiments in the ‘real world’; the latter may take place in a laboratory setting or, so to speak, in the wild (in the following I will focus on laboratory experiments). The epistemic role of thought and computational experiments has been and still is contested, one of the issues being whether or not they lead to new knowledge about the world [see, for instance (Mach 1976 (1897); Kuhn 1977)]. This is not the case for experiments performed in the real world; the epistemic relevance of the outcomes of these experiments is not disputed. Another distinction, which cuts across this one, is between qualitative and quantitative experiments. Qualitative experiments show the existence of particular phenomena, such as the quantum-interference of electrons in the double slit experiment. Quantitative experiments focus on quantitative relations between physical quantities and generally make use of the principle of parameter variation (here we may think of Boyle’s experiments with gases to show the relation between pressure and volume that bears his name). Again another distinction is based on the relation between experiment and theory. Depending on whether an experiment is intended to explore phenomena without the guidance of theory or is intended to test a theory, experiments may be divided, only schematically of course, into exploratory and hypothesis testing.

There are at least two reasons for performing laboratory experiments in the natural sciences:

1. experiments enable the study of spontaneously occurring physical objects and phenomena under conditions that do not occur spontaneously in the world; and
2. in experiments conditions may be created for the occurrence of physical objects and phenomena that do not occur spontaneously in the world; these objects and systems can therefore be studied only under experimental conditions.

Thus, the experimenter creates the appropriate conditions for studying physical phenomena and objects and may even create those phenomena and systems themselves, again by creating the appropriate conditions for their occurrence. These conditions are human-made and therefore artificial; that, however, does not imply that the physical objects and phenomena themselves are human creations and thus artificial (……).

What I am particularly interested in here is to what extent the experimenter has control over the system on which the experiment is performed. In this respect there are significant differences between thought, computer and laboratory experiments. In thought experiments the physical system under study and the conditions under which it is studied are created in the imagination and the experimenter has in principle total control over the system and these conditions; (s)he is even in a position to study physical systems under conditions that cannot be realized in the world (for instance, by assuming the validity of imaginary physical laws). However, there are restrictions on what kind of systems can be studied fruitfully in thought experiments. These restrictions find their origin in the reasoning powers of the experimenter; it is, for instance, no use to perform thought experiments on systems that are so complex that it is not possible to draw any interesting conclusion about their behavior.

More or less the same applies to computer experiments (simulations). *Prima facie,* the experimenter appears to have almost unlimited freedom to define the target system and its conditions. However, also in this case there are restrictions; the freedom of the experimenter is not unlimited due to constraints imposed by the computational device (computer). The computational power of the device puts limits on the kinds of target systems that may be simulated and therefore puts limits on the control of the experimenter over the target system and its conditions. These limits on the experimenter’s control find their origin in the technological limits (related to hardware and software) of the computational device.

When it comes to laboratory experiments, technological constraints determine the control of the experimenter over what kind of system may be studied under what kind of experimental conditions. Now it is not possible to study objects or systems that contradict the laws of nature, as is the case for thought and computational experiments. In the limit case where the scientist has no control whatsoever over the object of study and the conditions under which it is studied (e.g. the occurrence of a supernova) the scientist is dependent on Nature to perform the ‘experiment’ so as to make it possible to study the system or phenomenon (see Morgan’s notion of Nature’s experiment (Morgan 2013). This does not mean that the scientist is condemned to the role of a totally passive observer and that no issues of control emerge. The observation of (naturally occurring) phenomena may involve all kinds of measurement equipment that have to be controlled during the observations.

Note that in these various kinds of experiments the control of the experimenter stretches no further than control over what kind of system is studied and the conditions under which it is to be studied. The experimenter does not have any control over the behavior of the system under those conditions, that is, s/he has no control over the outcome of the experiment.^1^ If that would be the case, the performance of the experiment would lose its rationale: why perform an experiment if the outcome can be controlled and consequently predicted with certainty.^2^

## Experiments and Control in the Social Sciences

Let us now move over to experiments in the social sciences.^3^ Walker and Willer (2007, p. 25) distinguish between two “fundamentally different types of experiments” in the (social) sciences, namely empiricist and theory-driven experiments, each of which has its own method. This distinction runs more or less parallel to the above distinction between exploratory and hypothesis testing experiments. Empiricist experiments aim at making generalizations from observations, whereas theory-driven experiments aim at testing theories. The examples of experiments they discuss all fall into the above category of laboratory experiments. They do not refer to thought experiments or computational experiments; it appears that at least the latter also have come to play an important role in the social sciences. In the following I will focus on laboratory experiments only.

What emerges from the discussion of laboratory experiments in the social sciences in part I of Webster and Sell (2007a) is that it is all about control. According to Webster and Sell (2007b, p. 8) “a study is an *experiment* only when a particular ordering occurs: when an investigator controls the level of independent variables *before* measuring the level of dependent variables.” Walker and Willer (2007, p. 25) state that a laboratory experiment is “an inquiry for which the investigator plans, builds, or otherwise controls the conditions under which phenomena are observed and measured…”. According to Thye (2007, p. 66) “The confluence of three features makes experimental research unique in scientific inquiry: random assignment, manipulation, and controlled measurement.” All of these three features, including random assignment, are about control; the notion of the control group is based on randomization and the “first and most straightforward type of control is the *control group*” (Thye 2007, p. 79). Random assignment of persons in the experimental and control group is intended to eliminate (which is a specific form of control) the influence of spurious variables. This focus on controlling variables in laboratory experiments is of course closely related to the complexity of social phenomena in the sense of the number of potentially relevant variables for the phenomenon under study. In order to be able to draw reliable conclusions about the relation between the independent variables and the dependent variables all other variables that may have an influence on the dependent variables have to be kept constant.

The control of social phenomena in laboratory experiments has advantages and disadvantages. According to Webster and Sell (2007b, p. 11):

“The greatest benefits of experiments reside in the fact that they are artificial. That is, experiments allow observation in a situation that has been designed and created by the investigators rather than one that occurs in nature. Artificiality means that a well-designed experiment can incorporate all the theoretically presumed causes of certain phenomena while eliminating or minimizing factors that have not been theoretically identified as causal.”

Because the conditions are controlled, experiments may be replicated in order to test the “internal” validity of the outcomes. The disadvantage of artificiality is that it raises issues about generalizability and “external” validity: to what extent may the results from experiments be generalized to situations outside the laboratory settings? Internal and external validities are more or less in tension with each other: the more the conditions in the laboratory are artificial, that is, are different from the conditions in natural settings, the better it is for establishing interval validity, but the more problematic it may be for external validity (that is, for situations outside the laboratory) (Walker and Willer 2007, p. 51).

## The Traditional Control Paradigm for Experiments

If we compare the nature and role of experiments in the natural and social sciences, it is interesting to note, for a start, that Walker and Willer explicitly state (p. 52) that “theory-driven experiments and the application of well-supported theory outside the lab are exactly the same in the physical and social sciences.” I will not enter into a discussion whether or not this is indeed the case. Suffice it here to remark that *discussions* about the nature and role experiments in the natural and social sciences are not conducted on “exactly the same” lines; whereas issues about artificiality and internal and external validity play a prominent role in discussions about experiments in the social sciences, they are virtually absent in the natural sciences.^4^ Whether or not this meta-level difference is due to differences in the ‘logic’ or method of experiments in both cases or is due to difference in features of the subject matter studied (e.g., the ‘complexity’ of the subject matter) in the experiments remains to be seen.

Here I will focus on a feature that experiments in the natural and social sciences appear to have in common, namely their focus on control. In this respect there seems to be no significant difference between experiments in the natural and the social sciences. In both cases the experimenter strives for (total) control over the experiment. But what does the expression “control over the experiment” mean? In order to clarify this we have to carefully distinguish between two different forms of control. One form concerns control over the kind of system on which experiments are to be performed. In laboratory settings the kind of experimental system is usually designed and created by the experimenter; taking into account technological, social/ethical and other kinds of constraints she decides on the basis of her research interests how to configure the system on which the experiments will be performed. The aim of the experiment is to study the behavior of this particular kind of experimental system, which means that during an experiment the system itself has to remain the ‘same’, that is, has to remain an instance of that kind of experimental system. So, once an experiment has been defined and actually started the experimental system is no longer to be changed. This means that the experimental system is no longer under control of the experimenter in the sense that any change in that kind of system implies either a termination of the original experiment and the start of a new kind of experiment, or simply a termination of the original experiment.^5^ However, this does not preclude that the experimenter may have full control over when to start and when to terminate a particular experiment.

The other form of control pertains to the conditions under which the experimental system is to be studied, in particular to the interaction between the experimental system, once it is put in place, and its environment. This kind of control may take different forms. For instance, the experimenter may be interested in how the experimental system, starting from a given initial state, behaves in isolation from its environment. In that case, the experimenter brings the experimental system in the initial state through controlled interaction between the system and its environment and once it is in this state she closes off the system from all relevant interaction with the environment.^6^ In experiments with independent and dependent variables, the experimenter sets the independent variable through controlled interaction with the (environment of) the experimental system. Finally, in experiments involving control groups, the spurious variables not under direct control are controlled by following statistical procedures in composing the experimental and control groups (this is an aspect of the set-up of the experimental system itself)^7^ and by assuming that the interaction of the two groups with their environments during the experiment is the same *except for* the controlled intervention in or treatment of the (experimental) group. Under those conditions it is possible to apply a statistical variant of Mill’s method of difference and conclude that the observed (statistical) difference in the dependent variable between the experimental and control group is the effect of the controlled intervention/treatment.^8^

The basic assumption underlying the above ideas about control of experiments, both in the natural and social sciences is that the experimenter may control the experiment either through intervention in the environment of the experimental system or through intervention in the experimental system itself. The notion of intervention has a clear meaning: the experimenter is not part of the system on which the experiment is performed nor of its environment. The experimenter operates from a center of control which is not part of (‘outside’) the experimental system and its environment. From this vantage point the experimenter controls the experimental system and its interaction with its environment, which means that she has control over setting the independent variables and over starting/terminating the experiment. I will refer to this idea as the *traditional control paradigm for experiments*.

Of course, the idea of full control underlying this traditional control paradigm is based on an idealized view of experiments. In actual scientific practice it may be difficult to realize full control of experiments, in the two senses discussed above. Even in well-prepared physical experiments within the “secure” walls of laboratories it may be difficult to eliminate (control) all interfering interactions. The ideal is to eliminate interferences as much as possible in order to “purify” the phenomenon under study. If interferences cannot be fully eliminated, then it is very often still possible to take the effect of the still occurring interferences into account in interpreting the results of the experiment. The idea of full control becomes even more problematic in the case of field experiments. According to Schwarz and Krohn (2011, p. 123) “perhaps the most striking feature of field experiments is that they deal with objects “outside,” in an uncontrolled environment.” Field experiments, in agriculture for instance, are often performed precisely because one is interested to learn more about the behavior of the experimental object/phenomenon under uncontrolled conditions.^9^ This means that in field experiments the experimenter tries to expose the experimental object/phenomenon to the conditions in the “wild”. This, paradoxically, presupposes a form of control on the part of the experimenter, namely to maintain conditions that correspond as much as possible to the conditions in the wild. Whenever necessary or desirable it may be possible, moreover, to register/measure and (partly) control the uncontrolled interactions in order to analyze their effect on the experimental system. The fact that field experiments are conducted in an uncontrolled environment does not *per se* imply that this environment is uncontrollable.

So, in actual experimental practice it may be difficult and/or not desirable to exercise full control over the experiment. This, however, does not affect the idea that the experimenter may intervene in the experiment and that the notion of an intervention is well-defined. In the next section we will analyze some problems with the control paradigm in the case of new technologies as experiments that do undermine the notion of an intervention by the experimenter.

## Problems with the Control Paradigm

For a start, let us briefly look at a specific kind of intervention, namely stopping an experiment; at the beginning of this paper we mentioned the possibility of stopping an experiment as one of the reasons for treating new technologies as experiments. Strongly associated with the traditional control paradigm, or even as part of it, comes the idea that the experimenter is able to terminate an experiment when deemed necessary for whatever reason. I will now examine this idea in more detail, in particular how this idea plays out in the case the introduction of new technologies into society are treated as experiments.

First of all, note that stopping an experiment (of whatever kind) does not mean that it is always possible to restore the experimental system to its initial state. The experiment may bring about irreversible changes in the experimental system (think of experiments to test the buckling load of a beam). With regard to the introduction of new technologies as experiments this means that even if it is assumed that such an experiment may be stopped, it may bring about irreversible changes in the experimental system, in particular, irreversible social changes. This possibility of the occurrence of irreversible changes clearly will have to be taken into account in discussions on the moral acceptability of the new technology.

Second, as noted earlier, the experimenter has no control over the outcome of the experiment, that is, over the *behavior* of the experimental system under given conditions. Full control over these conditions and over the set-up of the experimental system does not exclude the occurrence of (totally) unexpected behavior of the experimental system. Especially when experiments are performed for the first time unexpected behavior may occur; the more an experiment becomes stabilized the better the behavior of the experimental system may be “under control” in the sense of being reliably and accurately predictable. With regard to the effect of unexpected behavior on the control over the experiment in the senses discussed above schematically the following two situations may be distinguished: the unexpected behavior does not or does affect the experimenter’s control over the experiment, in particular her ability to stop the experiment. In the first case, the experimenter stays in control and the unexpected behavior may or may not be a reason to end an experiment prematurely (think of the Stanford prison experiment that was stopped prematurely for moral reasons). In the second case, the experiment may get ‘out of control’ because the unexpected behavior of the experimental system may disrupt the control mechanisms used by the experimenter. So, the experimenter may lose the possibility of stopping an experiment or to control the effects of the unexpected behavior of the experimental system (think of the experimental test that led to the Chernobyl disaster). I see no reason why a similar situation could not occur with regard to societal experiments with new technologies. However, apart from a loss of control due to unexpected behavior, there is yet another reason why control in those experiments may be problematic. This brings me to my next point.

Third, societal experiments with new technologies involve experimental systems of a special kind, namely socio-technical systems. In my opinion the notions of an intervention and of a center of control become problematic when dealing with experiments on socio-technical systems. Examples of (complex) socio-technical systems are infrastructural systems such as electric power supply systems or public transport systems. The behavior of these systems is significantly affected by their technical components but the functioning of the systems as a whole depends as much on the functioning of these technical components as on the functioning of its social components (legal systems, billing systems, insurance systems etc.) and the behavior of human actors. Within socio-technical systems the technological and social subsystems have to be attuned to each other in order for such systems to operate successfully. Socio-technical systems are hybrid systems consisting of elements of various kinds, such as natural objects, technical artefacts, human actors and social entities like organizations and the rules and laws governing the behavior of human actors and social entities.

Elsewhere (…..) I have argued that the traditional engineering *design* paradigm is no longer a suitable basic framework for the design and control of socio-technical systems. This design paradigm is based on the following three pillars. First, it assumes that it is possible to clearly separate the object of design from its environment. Second, it deals exclusively with the designing of hardware (the manual is more or less taken for granted). What is designed is a material technical object. Third, it assumes that the behavior of the systems designed can be fully controlled by controlling the behavior of its parts. Given that the technical artifact is made up of physical parts, this control amounts to the control of the behavior of these physical parts through a set of control parameters. Similar to the control paradigm for experiments, the traditional design paradigm is based on the assumption that the designer operates from a control center outside the system to be designed.

For socio-technical systems the assumptions underlying the traditional design paradigm do not apply and the same reasons that undermine the traditional engineering design paradigm for these systems also undermine the traditional control paradigm for experiments with socio-technical systems. To begin with, there is the problem of where to draw the line between the experimental system and its environment. If the function of a system is taken to be that which gives the system cohesion, then it is rather obvious that all elements relevant to the functioning of a system should be included. But how is the function of, for instance, an electric power supply system to be defined? Different actors may have different views on this and may therefore have different opinions on what constitutes part of the experimental system and what belongs to its environment. In whatever way the boundaries of the experimental system will be drawn, it is clear that, since we are dealing with socio-technical systems, by definition human agents and social institutions will be integral parts of the experimental system. This means, secondly, that the nature of the experimental system to be controlled changes. Its inner environment no longer consists of material objects only. The control of these systems not only involves the control of technical but also of social elements. However, the behavior of human agents and social institutions cannot be controlled in the same way that the behavior of technological systems can be controlled. In so far as human agents perform certain operator roles in socio-technical systems one may try to control their behavior explicitly by fixing their behavior in terms of protocols that they have to follow in their operator roles. Or one may try to control their behavior in more implicit ways by conditioning their working environment such that it elicits various forms of (desired) behavior. Nevertheless, there appears to be an essential limit to these forms of control of the behavior of human agents in socio-technical systems, a limit that is related to the nature of human agency. The human agents who fulfill the operator roles in socio-technical systems remain *autonomous* agents whose behavior for *reasons of their own* may deviate from prescribed protocols in an uncontrollable way. For instance, operators may decide to start a strike in order to support demands by their labor union, or deviate from a protocol because following the protocol in a certain situation raises moral issues for the human agent/operator involved. Of course, the presence of this essential limit in controllability of socio-technical systems may raise important moral issues when performing experiments on this kind of systems.^10^ Finally, various actors from within the socio-technical system, with their own interpretations of the function of the system and their role in realizing it, may try to change, control or re-design parts of the system from within. As a result, the idea of controlling the experimental system from a control center outside it becomes highly problematic.

There is yet another feature of complex socio-technical systems that threatens the applicability of the traditional control paradigm for experiments with these systems, namely the possible occurrence of emergent phenomena. If we assume that emergent phenomena may occur during experiments with complex socio-technical systems, then they do pose a real challenge to the traditional control paradigm. The emergent behavior of a system cannot be reduced, more or less by definition, to the behavior of its constituent parts. This means that the behavior of the system as a whole cannot be completely controlled by controlling the behavior of its parts. So, emergence and control do not go hand in hand. According to Buchli and Santini (2005, p. 3) “there is a trade-off between self-organization [and emergence; P. K.] on one hand and specification or controllability on the other: if you increase the control over your system you will suppress self-organization capabilities.” Such a new trade-off principle would indeed constitute a significant break with the traditional design and control paradigms for socio-technical systems with emergent features. Recent blackouts in electricity systems are often mentioned as examples of emergent phenomena in complex socio-technical systems. If indeed they are, the social consequences of these blackouts clearly illustrate the possible moral impact of emergent phenomena in experiments with socio-technical systems.

The main conclusion to be drawn from the foregoing is that the notions of control of and intervention into the system on which the experiment is performed will lose its (standard) meaning when we are dealing with complex socio-technical systems. In my opinion this feature sets experiments on socio-technical systems apart from experiments in the natural and social sciences. That we are dealing here with different kinds of experiments is further supported by the fact that these experiments on socio-technical systems do not fit into the following table of experimental forms proposed by Morgan (2013, p. 342) (Table 1):

**Table 1:** Experimental forms

Experiment: control intervention	“Control” by nature/society	Scientist designs experiments to fit in with the world	Designed and controlled by scientist
“Intervention” by nature/society	Nature’s/society’s experiments	Natural experiments: events in the world post hoc reconstructed as such experiments	
Intervention by scientist		Field experiments: a priori designed for the world	Laboratory experiment

Roughly, Morgan’s notion of design and control pertains to what I have referred to so far as the control over the set-up of the experimental system and her notion of intervention to my notion of intervention in the sense of controlled interaction with the experimental system from its environment. As I have argued above, it is difficult to apply these notions to socio-technical systems and therefore experiments on this kind of system do not fit into Morgan’s table of experimental forms.

So, the outcome of our analysis is that if the introduction of new technologies in society is interpreted as an experiment, then from the point of view of control over the experiment we are dealing with a kind of experiment that is different from traditional experiments in the natural and social sciences. In closing, let me point briefly to a conception of experimentation that may be more appropriate to apply to the introduction of new technologies in society. It is discussed in Ansell (2012) who refers to this kind of experiments as ‘design experiments’. Here is how design experiments are characterized (Ansell 2012, pp. 163–164):

“Design experimentation starts with the presumption that the world is a messy place and that experiments will not be able to isolate the effects of single variables. In a design experiment, the experimenter presumes that the experiment will interact with the totality of the setting in which the experiment is conducted. The focus of a design experiment is not to definitely accept or reject a hypothesis, but rather to iteratively refine the intervention (design-redesign cycles). […] Design experiments do not create a sharp distinction between researchers and subjects; instead, the practitioners often become experimenters. […] In other words, design experiments do not fully control the conditions in which the experiment occurs, as laboratory experiments attempt to do.”

So, in design experiments there is no clear-cut distinction between the experimenter and the experimental system and nobody is in full control of the experimental system. Such a notion of experiment may be more fruitful when technological innovations are interpreted as social experiments. Ansell (2012, p. 172) remarks that “…this approach to experimentation loses the powerful mode of verification associated with controlled experiments (and for this reason, some might argue that it is not experimental at all).” Apart from the loss of control, however, there is yet another reason to question whether design experiments are experiments at all. The focus of a design experiment is to redesign the intervention on the fly. In the case of the introduction of technological innovations in society this means redesigning the technological innovation (and possibly redesigning its social context). In my terminology that amounts to changing the kind of system on which the experiment is performed. However, by changing the experimental system during the experiment it is no longer clear what kind of experiment is being performed, which means that it is no longer clear what is learned about what during the experiment.

The foregoing analysis of treating technological innovations as experiments on society is intended as a prolegomenon to discussions of the moral aspects of such experiments that take the idea of the analogy with experiments in the natural and social sciences seriously. I have tried to show that this analogy is rather problematic, at least from the point of view of controlling the experimental system. If that is indeed the case, caution is needed with the transfer of moral principles developed for experiments in the natural and social sciences (such as informed consent) to technological innovations conceived of as experiments on society.

## Footnotes

1 Note that this may not be true for thought experiments. Think of Newton’s famous bucket experiment; Newton simply assumed what would be the outcome of his thought experiment, namely, that the surface of a bucket of water rotating in absolute space would be curved. Mach questioned whether that would really be the case. This feature of thought experiments is closely connected to their contested epistemic status mentioned above.

2 This relates directly to the remark above that the physical phenomena themselves, although created in the experiment, are themselves natural phenomena and not artificial.

3 The following is mainly based on the discussion of experiments in the social sciences in part I of *Laboratory Experiments in the Social Sciences* edited by Webster and Sell (2007a).

4 A notable exception is (Cartwright 1983).

5 Of course, this raises intricate problems about identity criteria for experimental systems and for experiments; in the present context the precise nature of these identity criteria, if there are any, is not of interest; what matters here is the contrast between control over the experimental system and control over the conditions under which experimental systems are studied.

6 Of course, any experiment involves making observations of (measurements on) the experimental system so it cannot be totally isolated from its environment.

7 ‘Control over the interaction’ here does not necessarily mean that the experimenter controls/knows who is part of the experimental group and who of the control group; in order to avoid experimenter’s bias it may be necessary to apply double blind procedures.

8 “If an instance in which the phenomenon under investigation occurs, and an instance in which it does not occur, have every circumstance save one in common, that one occurring only in the former; the circumstance in which alone the two instances differ, is the effect, or cause, or an necessary part of the cause, of the phenomenon.” John Stuart Mill, *A System of Logic*, Vol. 1. 1843. p. 455.

9 If indeed Schwarz and Krohn (2011, pp. 123–130) are right that important features of field experiments include “individuality, uniqueness, contingency, instability, and also potentially lack of safety”, then what is learned from field experiments may not be transferable and generalizable to other situations.

10 This essential limit in controllability, due to the fact that human beings are part of the experimental system, already manifests itself in experiments in the social sciences. The idea that the experimenter is not part of the environment of the experimental system may be difficult to realize in certain experiments in the social sciences, because subjects participating in an experiment may know that they do so and know that an experimenter is observing their behavior (i.e., is part of the environment) and this knowledge may influence their behavior in an uncontrollable way.
